# Validation of 10-Year Stroke Prediction Scores in a Community-Based Cohort of Chinese Older Adults

**DOI:** 10.3389/fneur.2020.00986

**Published:** 2020-10-22

**Authors:** Yanlei Zhang, Xianghua Fang, Shaochen Guan, Xiaoguang Wu, Hongjun Liu, Chunxiu Wang, Zhongying Zhang, Xiang Gu, Chunxiao Liu, Jianhua Cheng

**Affiliations:** ^1^Department of Neurology, the First affiliated Hospital of Wenzhou Medical University, Wenzhou, China; ^2^Evidence-based Medical Center, Xuanwu Hospital, Capital Medical University, Beijing, China; ^3^Geriatric Department, Xuanwu Hospital, Capital Medical University, Beijing, China; ^4^Geriatric Department, Friendship Hospital, Capital Medical University, Beijing, China; ^5^Geriatric Department, Beijing Geriatric Hospital, Beijing, China

**Keywords:** stroke, risk factors, prediction score, primary prevention, cohort study

## Abstract

A stroke prediction model based on the Prediction for Atherosclerotic Cardiovascular Disease Risk in China (China-PAR) project was developed. We compared its predictive ability with the revised Framingham Stroke Risk Score (R-FSRS) for 5-year stroke incidence in a community cohort of Chinese adults, namely the Beijing Longitudinal Study of Aging (BLSA). Calibration, discrimination, and recalibration were used to compare the predictive ability between the two prediction models. Category-less net reclassification improvement (NRI) and integrated discrimination improvement (IDI) values were also assessed. During a mean follow-up duration of 5.1 years, 106 incidents of fatal or non-fatal strokes occurred among 1,203 participants aged 55–84 years. The R-FSRS applied to our cohort underestimated the 5-year risk for stroke in men and women. China-PAR performed better than the R-FSRS in terms of calibration (men, R-FSRS: χ^2^-value 144.2 [*P* < 0.001], China-PAR: 10.4 [*P* = 0.238]; women, R-FSRS: 280.1 [*P* < 0.001], China-PAR: 12.5 [*P* = 0.129]). In terms of discrimination, R-FSRS and China-PAR models performed modestly in our cohort (C-statistic 0.603 [95% CI: 0.560–0.644] for men using China-PAR and 0.568 [95% CI: 0.524–0.610] using the R-FSRS; the corresponding numbers for women were 0.602 [95% CI: 0.564–0.639] and 0.575 [95% CI: 0.537–0.613). The recalibrated China-PAR model significantly improved the discrimination in C statistics and produced higher category-less NRI and IDI for stroke incidence than the R-FSRS. Although China-PAR fairly estimated stroke risk in our cohort, it did not sufficiently identify adults at high risk of stroke. Caution would be exercised by practitioners in applying the original China-PAR to Chinese older adults. Further studies are needed to develop an adequate prediction model based on the recalibrated China-PAR or to find new risk markers which could upgrade this model.

## Introduction

Stroke is the second most prevalent cause of death and disability (1), particularly in the aged population, which presents a large proportion of the Chinese population, with population aging having significantly increased stroke incidence (2). The prevalence and incidence of stroke have increased in China over the past 3 decades. Current statistics demonstrate that an estimated 2.4 million incidents of strokes occur annually, adding to the pool of 11.1 million stroke survivors who return to community after treatment at the acute stage (3). Thus, an effective screening tool is required for identifying adults at high risk of stroke for primary prevention, which might be the best choice for cost/benefit balance in managing stroke.

Several multivariate risk prediction models have been developed following the original Framingham Stroke Risk Score (FSRS), which is based on various vascular risk factors for predicting the risk of initial stroke (4–8). The FSRS has been the most widely used tool worldwide, and the revised Framingham Stroke Risk Score (R-FSRS), which is the most recent version to reflect the temporal trends, was published in 2017 (8). However, most prediction models have been developed based on Western populations. Therefore, applying these models to the Chinese population might not be appropriate. In 2019, Xing et al. (9) established a new stroke prediction model aimed at predicting stroke risk among Chinese individuals included in the Prediction for Atherosclerotic Cardiovascular Disease Risk in China (China-PAR) project. The China-PAR stroke risk models were developed based on a middle-aged cohort (40–79 years, mean age 48.6 years) and included factors such as age and age-related diseases. However, whether these findings are generalizable in elderly populations of the community is unknown.

The present study aimed to assess the external validity of the China-PAR stroke risk models in a community cohort of Chinese adults aged 55 years and over, namely the Beijing Longitudinal Study of Aging (BLSA), and to compare this prediction model with the R-FSRS.

## Materials and Methods

### Study Population

We used data from the BLSA to validate the R-FSRS and China-PAR 10-year stroke prediction models. The study design has already been reported elsewhere (10, 11). The BLSA was a prospective population-based cohort study to investigate health conditions in residents aged 55 years and over in Beijing, China. Briefly, a stratification-random clustering procedure was applied to the sample to ensure representativeness in terms of the average age, education, and economic level. The present study was based on the survey conducted in 2009. Among all 2468 participants aged 55 years and older, 2089 (84.6%) completed the follow-up survey with a mean follow-up time of 4.8 years.

The R-FSRS was developed to assess the risk of stroke in individuals aged 55–84 years with no history of stroke (8). The China-PAR was mainly developed based on Chinese individuals aged 35–74 years to predict the 10-year risk of stroke (12). In order to make the samples as comparable as possible, we excluded participants aged 85 years or older. Among all participants, 1,203 were eligible for analysis after excluding those with history of stroke (*n* = 297), aged over 84 years (*n* = 153), and with missing data on measurements of blood specimens (*n* = 815; Supplementary Figure 1).

### Risk Factors Measurement

All enrolled participants were asked to complete the baseline assessment, which consisted of answering a questionnaire and undergoing a physical examination, a fasting blood sample collection.

All enrolled participants were asked to complete a questionnaire survey conducted by well-trained medical students using standardized methods. The questionnaire covered a wide range of variables, including demographic characteristics; life-style habits (smoking and drinking status); history of stroke, heart disease, hypertension, diabetes, hyperlipidemia, and other chronic diseases related to aging; and the use of medication.

Before the examination, each subject was asked to rest for ≥20 min. The sitting blood pressure (BP) was measured twice on the right arm in 2–5-min intervals, and the mean of the two measurements was calculated and use for the analysis. BP was measured using a standard mercury sphygmomanometer. The measurements of height and body weight were also collected for each subject.

Blood samples were collected from the subjects in the morning after an overnight fast, centrifuged to collected the serum, stored in a refrigerator at 2–8°C, and transferred to a central laboratory (IPE Center for Clinical Laboratory, Beijing, China), which performed all analyses within 24 h. Total cholesterol, triglyceride, high-density lipoprotein cholesterol (HDL-C), low-density lipoprotein cholesterol, fasting blood glucose (FBG), and high-sensitivity C-reactive protein levels were determined by a Hitachi 7600 automatic analyzer (Hitachi High-Technologies Corporation, Tokyo, Japan).

### Follow-up and Study Outcomes

In December of 2014, a follow-up survey was performed to investigate the outcomes of each subject who underwent the 2009 baseline assessment. A door-to-door survey was conducted by well-trained graduate students of medicine. Information on possible new cases of stroke and deaths that occurred during the follow-up period was collected. The study subjects' medical and health insurance in local medical clinics were also reviewed by the physicians who participated in the follow-up survey. The local government death records kept at the Center for Disease Control (CDC), were also reviewed to collect the date and causes of death. In China, the CDC is in charge of death statistics, and the underlying causes of death are coded according to the principles of the 10th version of the International Classification of Diseases (ICD-10). Codes I63.0-I63.9 and I60.0-I62.9 represent fatal stroke. The study outcome was defined as stroke occurrence.

### Definitions

Hypertension was defined following the Joint National Committee guideline (JNC VII) (13) to include subjects with systolic BP (SBP) ≥ 140 mmHg or/and diastolic BP (DBP) ≥ 90 mmHg, or those with a history of hypertension or taking anti-hypertension medications. The diagnosis of diabetes was based on the American Diabetes Association criteria as FBG ≥ 7.0 mmol/L (126 mg/dL) (14), having a history of diabetes, or taking hypoglycemic medication. Smokers included current and ex-smokers for those who had quit smoking for > 2 years.

### Prediction Models

We calculated the stroke risk for each BLSA participant using the published models of the R-FSRS (15) and China-PAR 10-year stroke risk models (9). Supplementary Table 1 presents the predictor variables used in the R-FSRS and China-PAR models. Although family history of stroke was used in the China-PAR 10-year stroke risk model, it was not assessed in the BLSA. Therefore, for this factor, missing values were attributed to all participants. All participants in the BLSA data lived in northern China, and, therefore, no Cox hazard regression coefficient could be estimated. For these analyses, the 5-year baseline survival rate was replaced to predict the 5-year stroke risk based on the 10-year risk models.

To compare the performance of the two prediction models, three versions of each model were used to calculate the 5-year stroke risk for every participant (16). The three versions of the prediction models differed in terms of baseline survival rate, the means of the risk factors, andthe Cox hazard regression coefficients of risk factors. The original versions of the prediction models used these parameters based on the original research, thus possibly overestimating or underestimating the stroke risk of participants. Therefore, the adjusted versions could optimize data fit. We used the mean values of risk factors and baseline survival rate, which were derived from BLSA data in the adjusted versions. In theory, we could produce a suitable prediction model by adopting coefficients of risk factors to compensate for different background incidence rates in different populations (17). To represent the best possible risk function for BLSA data, we updated the mean values of risk factors to represent the current prevalence, updated the baseline survival rate of stroke to represent current rates, and updated cox hazard regression coefficients to represent current associations in the recalibrated prediction models. The cox hazard regression coefficients for components in the models were compared (Supplementary Tables 2, 3).

### Statistical Analysis

Risk factors are summarized for sex-specific groups as mean (SD) for continuous variables and percentile for categorical variables. Chi-square test or Fisher's exact test was used for comparing categorical variables, and the *t*-test or the Kruskal–Wallis test for comparing continuous variables. The Kaplan–Meier product-limit method was used to estimate the survival rate.

Calibration and discrimination were used to evaluate the predictive capabilities of all prediction models. Calibration, which measured how closely the predicted stroke risk fit the observed stroke risk, was assessed using the Hosmer–Lemeshow goodness-of-fit test. Values of χ^2^ more than 20 were considered to indicate significant lack of calibration (*P* < 0.01).

Discrimination of all prediction models was assessed using C statistics. Differences in C statistics between the two prediction models were evaluated using the method by DeLong et al. Category-less net reclassification improvement (NRI) and integrated discrimination improvement (IDI) values were also assessed (18).

## Results

### Baseline and Follow-Up Information

Of the 1,203 participants in our analysis, 537 (44.6%) were men; the mean age of participants was 68.6 years. The overall and sex-specific baseline characteristics of the participants selected for this analysis are summarized in Table 1. Compared with women, men were older and had lower total cholesterol values (all *P* < 0.05). The proportions of participants with diabetes and of those taking antihypertensive medication were smaller among men than among women, whereas the proportion of current smokers was substantially greater among the former (all *P* < 0.05).

**Table 1:** Baseline of characteristics of study participants by sex.

**Characteristics**	**Total**	**Men (*n* = 537)**	**Women (*n* = 666)**	***P*-value**
Age, mean (SD), year	68.63 (7.62)	69.21 (7.73)	68.15 (7.49)	0.016
Age ≥ 65, *n* (%)	795 (66.1)	366 (68.2)	429 (64.4)	0.102
SBP, mean (SD), mmHg	138.7 (19.99)	138.18 (19.58)	139.17 (20.31)	0.392
Waist circumference, mean(SD), cm	90.8 (10.67)	88.94 (9.73)	91.08 (10.69)	0.307
Total cholesterol, mean(SD), mg/dl	227.45 (44.12)	214.48 (39.01)	237.91 (45.24)	<0.001
HDL-C, mean (SD), mg/dl	47.38 (10.60)	46.72 (10.99)	47.90 (10.24)	0.054
Urban, *n* (%)	800 (66.5)	362 (67.4)	438 (65.8)	0.58
Antihypertensive treatment, *n* (%)	464 (38.6)	171 (31.8)	293 (44.0)	<0.001
Diabetes, *n* (%)	194 (16.1)	73 (13.6)	121 (18.2)	0.033
DM if <65 year, *n* (%)	54 (4.5)	18 (3.4)	36 (5.4)	0.094
DM if ≥65 year, *n* (%)	140 (11.6)	55 (10.2)	85 (12.8)	0.205
Smoking, *n* (%)	296 (24.6)	246 (45.8)	50 (7.5)	<0.001
History of CVD, *n* (%)	187 (15.5)	77 (14.3)	110 (16.5)	0.337

*SBP, systolic blood pressure; HDL-C, high-density lipoprotein cholesterol; DM, diabetes mellitus; CVD, cardiovascular disease*.

By the end of 2014, follow-up was conducted for a total of 6,139 person-years.

During a mean follow-up duration of 5.1 years, 106 incidents of fatal or non-fatal strokes (in 50 men and 56 women) occurred. The follow-up rate was 87.5%, with 150 participants lost to follow up. The 5-year cumulative incidence of stroke was 1,851.9 per 100,000 person-years and 1,628.3 per 100,000 person-years in men and women, respectively.

### Calibration

**Figure 1:**
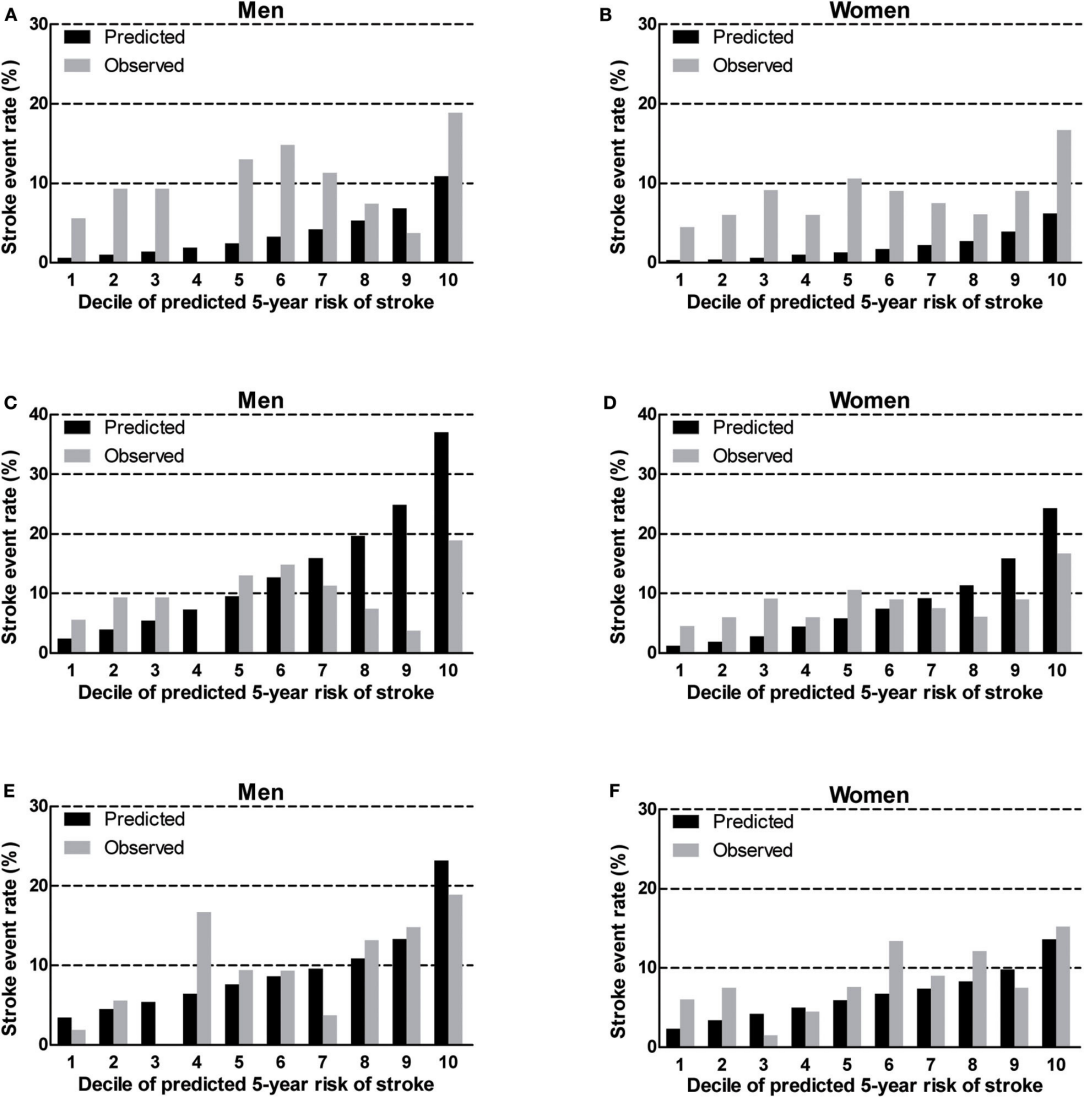
Calibration of the original R-FSRS **(A,B)**, adjusted R-FSRS **(C,D)**, and recalibrated R-FSRS **(E,F)** in BLSA men and women. R-FSRS, revised Framingham Stroke Risk Scores; BLSA, Beijing Longitudinal Study of Aging.

**Figure 2:**
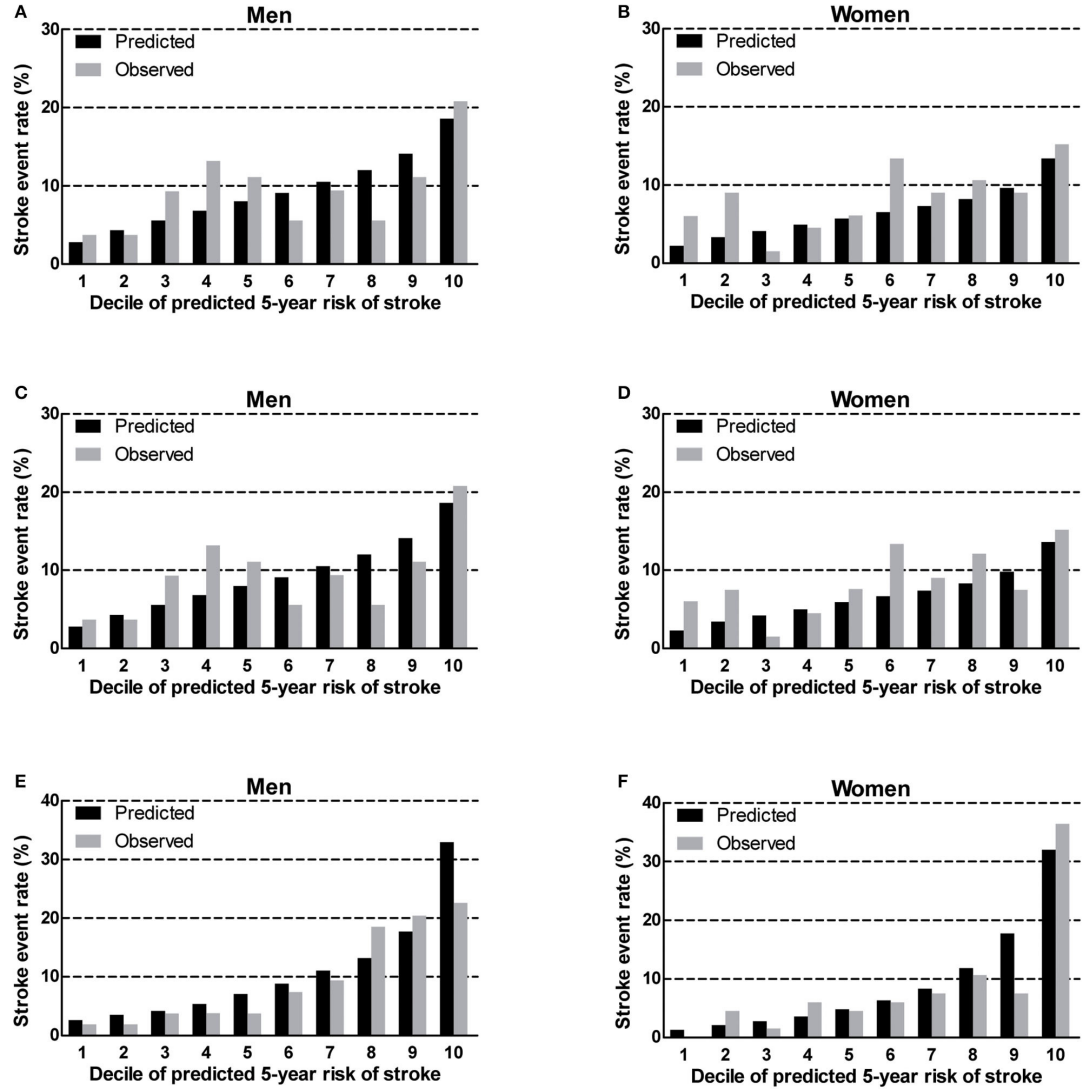
Calibration of the original China-PAR **(A,B)**, adjusted China-PAR **(C,D)**, and recalibrated China-PAR **(E,F)** in BLSA men and women. China-PAR, Prediction for ASCVD Risk in China; BLSA, Beijing Longitudinal Study of Aging.

We found that the China-PAR performed better than the R-FSRS in terms of calibration (men: R-FSRS: χ^2^ value 144.2 [*P* < 0.001], China-PAR: 10.4 [*P* = 0.238]; women: 280.1 [*P* < 0.001], and 12.5 [*P* = 0.129]). The calibration plots indicated that the R-FSRS underestimated the expected stroke rate among men and women compared to the observed rates (Figures 1A,B). Compared with the original R-FSRS, the adjusted R-FSRS showed improved model calibration (men: χ^2^ value 39.5 [*P* < 0.001]); women: χ^2^ value 31.7 [*P* < 0.001]); however, this model still slightly overestimated stroke risk among men and underestimated stroke risk among women (Figures 1C,D). Calibration plots (Figures 1E,F) showed that the recalibrated R-FSRS model did not underestimate or overestimate stroke events among men and women (all calibration χ^2^ values <20). As also shown in Figure 2, both the adjusted and recalibrated China-PAR performed well in the calibration analysis (all calibration χ^2^ values <20). In addition, the recalibrated China-PAR had the lowest calibration χ^2^ values in all prediction models.

### Discrimination

**Figure 3:**
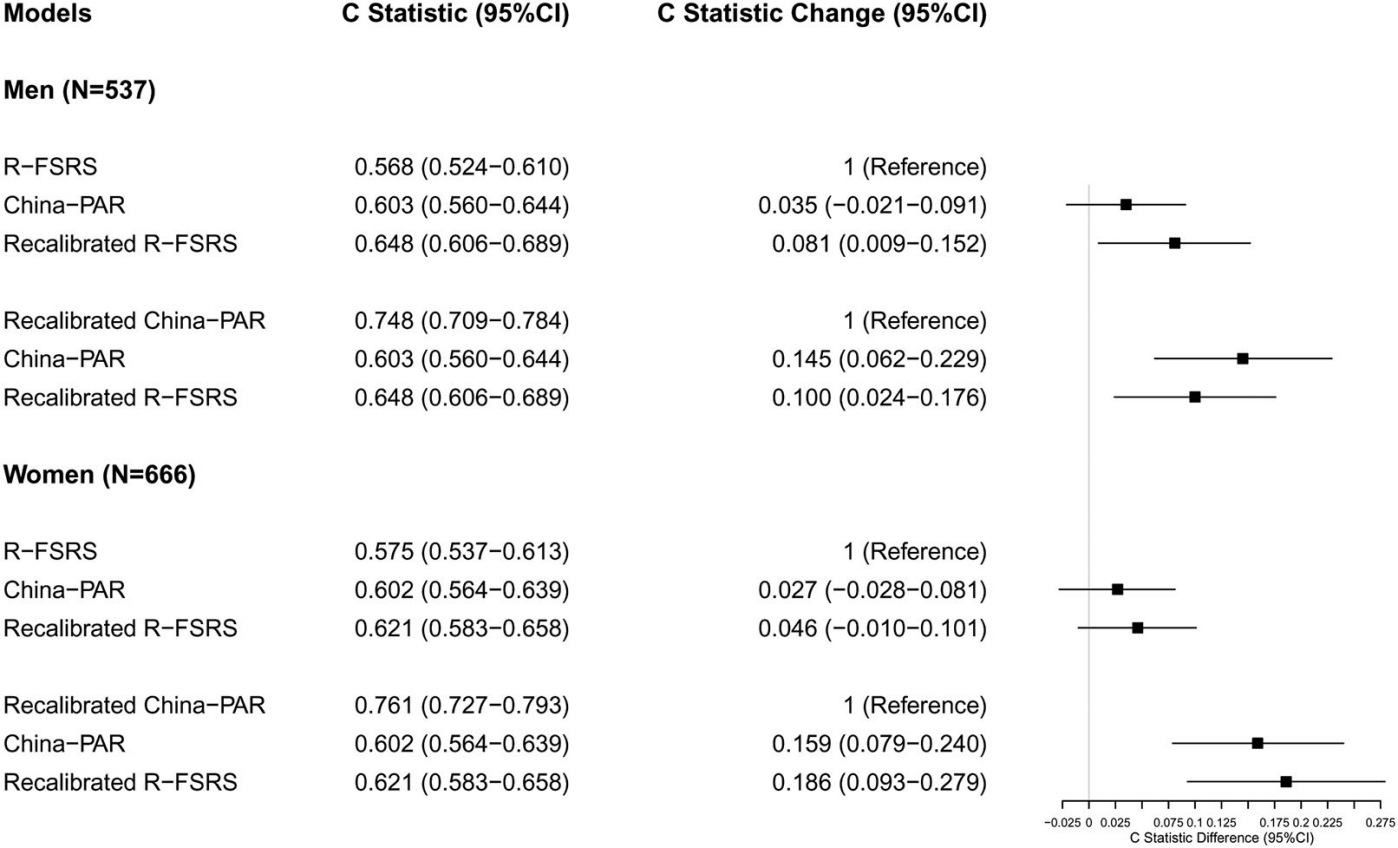
Differences in C statistics between the China-PAR, R-FSRS, and recalibrated prediction models in BLSA men and women. R-FSRS, revised Framingham Stroke Risk Scores; China-PAR, Prediction for ASCVD Risk in China; BLSA, Beijing Longitudinal Study of Aging.

As shown in Table 2, the R-FSRS and China-PAR models performed modestly in our cohort (C statistic: 0.603 [95% CI: 0.560–0.644] for men using China-PAR and 0.568 [95% CI: 0.524–0.610] using the R-FSRS; the corresponding values for women were 0.602 [95% CI: 0.564–0.639] and 0.575 [95% CI: 0.537–0.613]). C-statistic values of the adjusted China-PAR and R-FSRS were similar to those of the original models. Figure 3 displays the differences in C statistics between the China-PAR, R-FSRS, and recalibrated prediction models. There were no significant differences in C statistics for stroke between the China-PAR and R-FSRS for both sexes. The performance of the recalibrated China-PAR model was excellent as suggested by C statistics (men: 0.748 [95% CI: 0.709–0.784]; women: 0.761 [95% CI: 0.727–0.793. According to the China-PAR model, the recalibrated China-PAR showed a significant improvement in performance C statistics from 0.603 to 0.748 (C statistics difference: 0.145, 95% CI: 0.062–0.229) in men and from 0.602 to 0.761 (C statistics difference: 0.159, 95% CI: 0.079–0.240) in women. In contrast, C statistics for the performance of the recalibrated R-FSRS for men improved from 0.568 to 0.648 (C statistics difference: 0.081, 95% CI: 0.009–0.152). Although the R-FSRS and China-PAR models both updated the mean values of risk factors, survival rate of stroke, and cox coefficients in order to represent the best possible risk function for BSLA data, the recalibrated China-PAR could better identify individuals at a high risk of stroke than did the recalibrated R-FSRS (men: C statistics, 0.748 vs. 0.648, C statistics difference: 0.100, 95% CI: 0.024–0.176; women: C statistics: 0.761 vs 0.621,; C statistics difference: 0.140, 95% CI: 0.051–0.230). There was no significant difference in C-statistic values between the recalibrated and original R-FSRS (C statistics: 0.621 vs. 0.575, C statistics difference: 0.046, 95% CI: −0.010 to 0.101) in women.

**Table 2:** Validation of 5-year stroke risk prediction by the three versions of R-FSRS and China-PAR in men and women.

**Models**	**Kaplan–Meier adjusted events (*n*)**	**Predicted Events (*n*)**	**Calibration χ^2^ values**	***P*-value**	**Discrimination C statistic (95%CI)**
**MEN**					
R-FSRS	54.8	20.3	144.166	<0.001	0.568 (0.524–0.610)
Adjusted R-FSRS	54.8	74.2	39.493	<0.001	0.568 (0.524–0.610)
Recalibrated R-FSRS	54.8	49.7	16.481	0.036	0.648 (0.606–0.689)
China-PAR	54.8	43.3	10.405	0.238	0.603 (0.560–0.644)
Adjusted China-PAR	54.8	49.2	9.375	0.312	0.603 (0.560–0.644)
Recalibrated China-PAR	54.8	60.87	6.334	0.610	0.748 (0.709–0.784)
**WOMEN**					
R-FSRS	59.9	13.6	280.054	<0.001	0.575 (0.537–0.613)
Adjusted R-FSRS	59.9	55.98	31.743	<0.001	0.575 (0.537–0.613)
Recalibrated R-FSRS	59.9	43.36	11.926	0.155	0.621 (0.583–0.658)
China-PAR	59.9	69.7	12.524	0.129	0.602 (0.564–0.639)
Adjusted China-PAR	59.9	44.56	16.047	0.042	0.604 (0.566–0.642)
Recalibrated China-PAR	59.9	60.3	9.796	0.280	0.761 (0.727–0.793)

*R-FSRS, revised Framingham Stroke Risk Scores; China-PAR, Prediction for ASCVD Risk in China*.

The category-less NRI for China-PAR, R-FSRS, and their recalibrated versions were calculated from the difference in the estimated risk of stroke moving up and down among these prediction models (Table 3). Compared with the R-FSRS model, the China-PAR model could not improve risk classification for the 5-year stroke risk, as evidenced by the category-less NRI in men (0.004 [95% CI: −0.004 to 0.012]) and in women (0.007 [95% CI: −0.003 to 0.016]). However, the IDIs were improved both in men (0.018 [95% CI: 0.0020–0.033]) and in women (0.014 [95% CI: 0.001–0.027]). Similar results were observed when comparing the recalibrated with the original R-FSRS. The recalibrated China-PAR models were more likely than the original China-PAR models to provide higher category-free NRI among men (0.617 [95% CI: 0.342–0.894]) and women (0.611 [95% CI: 0.344–0.879]). This discrimination improvement was also confirmed by IDI among men (0.056 [95% CI: 0.023–0.090]) and women (0.092 [95% CI: 0.054–0.130]). Reclassification analyses also showed higher category-free NRI and IDI for 5-year stroke incidence when comparing the recalibrated China-PAR to the recalibrated R-FSRS models.

**Table 3:** Category-less NRI and IDI between R-FSRS, China-PAR, and their recalibrated versions.

	**Men**		**Women**	
	**Event**	**Nonevent**	**Event**	**Nonevent**
FSRS > China-PAR (Downward)	0	1	0	2
FSRS < China-PAR (Upward)	50	486	56	608
Overall	50	487	56	610
NRI	1	−0.996	1	−0.993
Total NRI (95%CI)	0.004 (−0.004–0.012)		0.007 (−0.003–0.016)	
IDI (95%CI)	0.018 (0.0020–0.033)		0.014 (0.001–0.027)	
R-FSRS > Recalibrated R-FSRS (Downward)	1	17	2	3
FSRS < Recalibrated FSRS (Upward)	49	466	54	606
Overall	50	483	56	609
NRI	0.960	−0.922	0.928	−0.989
Total NRI (95%CI)	0.038 (−0.047–0.123)		−0.058 (−0.156–0.040)	
IDI (95%CI)	0.027 (0.007–0.048)		0.008 (0.001–0.016)	
China-PAR > Recalibrated China-PAR (Downward)	17	312	23	433
China-PAR < Recalibrated China-PAR (Upward)	33	167	33	167
Overall	50	479	56	610
NRI	0.320	0.297	0.179	0.436
Total NRI (95%CI)	0.617 (0.342–0.894)		0.611 (0.344–0.879)	
IDI (95%CI)	0.056 (0.023–0.090)		0.092 (0.054–0.130)	
Recalibrated FSRS > Recalibrated China-PAR (Downward)	2	61	16	331
Recalibrated FSRS < Recalibrated China-PAR (Upward)	48	421	40	276
Overall	50	482	56	610
NRI	0.920	−0.739	0.429	0.090
Total NRI (95%CI)	0.175 (0.051–0.298)		0.517 (0.268–0.767)	
IDI (95%CI)	0.074 (0.040–0.108)		0.098 (0.159–0.137)	

*IDI, integrated discrimination index; NRI, net reclassification improvement; R-FSRS, revised Framingham Stroke Risk Scores; China-PAR, Prediction for ASCVD Risk in China*.

## Discussion

The China-PAR stroke risk prediction model is the most recent tool to identify high-risk stroke adults in China (9). The validity of this risk model among samples of different age ranges has not been studied. To our knowledge, no previous study has compared the performances of the R-FSRS and China-PAR 10-year stroke risk models for the 5-year risk of stroke in a prospective community-based cohort of residents aged 55–84 years without known stroke at baseline. Compared with the R-FSRS model, the China-PAR model showed a better calibration and similar discriminative ability for the 5-year risk of stroke in our cohort. In contrast, the R-FSRS model underestimated the 5-year stroke. The recalibration analysis significantly improved the discriminative ability of China-PAR.

The FSRS, which is widely used for primary prevention of stroke, was established in 1991 to evaluate the 10-year risk of stroke in the western population aged 55–84 years (19). The FSRS was updated as R-FSRS to reflect the present situation of risk factors of stroke (8). Several studies have revealed conflicting results when applying these predictive models to different cohorts. The Reasons for Geographic and Racial Differences in Stroke study found that the use of the FSRS leads to overestimation in predicting stroke risk among black and white participants (20). An overestimation was also reported in the Three-City study, which validated the FSRS among French adults aged 65 years or older (16). The FSRS and R-FSRS were predictive of incident stroke in the Rotterdam study, which included 7966 stroke-free subjects (15). Similar results were also observed when evaluating stroke risk among participants of the Multi-Ethnic Study of Atherosclerosis (21). There are limited studies showing different results regarding the performance of these stroke risk models among Chinese individuals. The CIMIC study validated the R-FSRS among 34,357 Chinese participants aged 55-74 years at baseline recruited between 2007 and 2008 (9). The result revealed that discrimination was moderate based on C statistics (men: 0.668; women: 0.686), while calibration was poor in men (χ^2^ value: 120.3) and women (χ^2^ value: 123.7). The 5-year risk of stroke was underestimated by 43.1% for men and 50.7% for women. An underestimation of stroke risk using the R-FSRS model was also noted in the current study. However, recalibration analysis by BLSA data corrected this underestimation. This might be attributed to ethnic heterogeneities, distinct risk factors of stroke burden, and different treatment rates for risk factors between Chinese and Western populations (22–25). Furthermore, differences in the stroke incidence rate could be the direct factor explaining this result. In China, the age-standardized stroke incidence rate, estimated at 247 per 100,000 person-years, suggests that the absolute stroke incidence rate in China is the highest in the world (3).

Our results indicate that the calibration χ^2^ value is lower in the case of China-PAR than of the R-FSRS. In contrast, the China-PAR model may not have sufficient discriminatory ability (C statistics) to identify individuals at high risk of stroke in our cohort. Several factors might explain this moderate discriminatory ability. First, although the China-PAR model was developed in 2019, data on the derivation cohort were collected in 1998, whereas BLSA data were gathered in 2008. In fact, the predictive model might be slightly outdated because the management of stroke risk factors has considerably evolved during the past 20 years. Second, BLSA participants were older than China-PAR participants (68.6 vs. 48.6 years). In addition, BLSA participants had a higher mean SBP, higher total cholesterol levels and waist circumference; had lower mean HDL-C levels; used more antihypertensive medication; were more often smokers; and more of them had diabetes. Third, the R-FSRS was derived and validated in participants without stroke at baseline. Thus, the present study excluded participants with a history of stroke, and, since the China-PAR was established in cohorts without history of myocardial infarction (MI) and stroke, this could have influenced its performance. It thus may be unfair to compare the China-PAR with the R-FSRS in our cohort. Nevertheless, prevalent cardiovascular disease is a risk factor included in the R-FSRS. Moreover, both these prediction models consider stroke as their primary endpoint and were established for predicting personalized stroke risk among adults free of MI and stroke (China-PAR) or adults free of stroke (R-FSRS). Therefore, it is reasonable to compare the discriminative ability of the China-PAR and the R-FSRS for stroke incidence in the primary prevention cohort.

Compared with the China-PAR study, the follow-up duration of the BLSA was relatively shorter. Moreover, the C statistic of the China-PAR model for predicting the 5-year risk of stroke was reduced from 0.792 to 0.716 in men and from 0.802 to 0.715 in women among participants aged 55 years and older in the validation cohort of the China-PAR study (9). The recalibrated China-PAR model predicted a stroke incidence rate that was reasonably close to the observed one and meaningfully improved the ability to identify individuals at high risk of developing stroke in the future among BLSA participants. This also suggests that the recalibrated China-PAR model could be extended to apply to individuals aged 55-84 years in northern China. These findings suggest that a recalibration analysis reflecting the characteristics of the current population might be an effective solution. Moreover, the original prediction models should be developed for similar populations, in terms of same race, lifestyle, and so on. However, most clinicians might have trouble to recalibrate prediction risk models for their population. Therefore, caution should be exercised by practitioners when applying the original China-PAR model to Chinese older adults, as its ability to identify individuals at high risk of stroke is insufficient. Therefore, additional risk factors of stroke risk besides age are needed in Chinese older adults. Markers of atherosclerosis might be a choice (21, 26–28). Further work should be done to evaluate the value of such makers in improving the discriminative ability of models to predict stroke even in older populations.

The current study has several strengths, including the relatively large sample size, representativeness of local residents, and standardized processes for collecting baseline and follow-up information. Our study contains the following limitations. First, nearly one third of the participants in the BLSA cohort had missing data on laboratory measurements, which is common in cohort studies (12, 29). However, compared with individuals with missing data, those with complete data were younger and fewer of them had DM, which might have led to an underestimation of the prediction scores (Supplementary Table 4). Second, participants with atrial fibrillation (AF) and parental history of stroke were not included in our cohort, and these parameters were set to 0 when calculating risk by risk models; thus the risk of stroke might be underestimated. However, such an impact could be limited due to the low prevalence of AF among Chinese individuals (30). A parental history of stroke has been suggested as a predictor of stroke events (31). Nevertheless, it was used in the China-PAR stroke risk model only for men, while the predictive ability could be slightly influenced in men in our cohort, and it is a stronger risk factor in early onset stroke (age <55) (32, 33); thus, it may not be so important that participants without such history are missing from our cohort. Third, the adjudication of stroke fatality using ICD coding on death certificates may have good specificity but likely suboptimal sensitivity (34). Finally, due to the relatively short follow-up period, the modest discrimination ability of the prediction models in our cohort should be interpreted with caution. Additional research is required to confirm whether the performance of these prediction models could be improved with longer follow-up duration.

## Conclusion

The present study revealed that the R-FSRS underestimates the 5-year absolute risk of stroke in a community-based Chinese population aged 55–84 years. Regardless of the fact that the China-PAR fairly predicted the risk of stroke, it showed a modest discrimination ability for incident of stroke. The recalibration process reasonably improved the discrimination ability of China-PAR. Further studies are needed to develop an adequate prediction model based on the recalibrated China-PAR and to identify new risk markers which could upgrade this model.

## Data Availability Statement

The datasets generated for this study are available on request to the corresponding author.

## Ethics Statement

The studies involving human participants were reviewed and approved by Ethical Committee of Xuanwu Hospital, Capital Medical University, Beijing, China. The patients/participants provided their written informed consent to participate in this study. Written informed consent was obtained from the individual(s) for the publication of any potentially identifiable images or data included in this article.

## Author Contributions

XF, SG, and JC contributed conception and design of the study. HL, CW, ZZ, XG, and CL organized the database. YZ and XW performed the statistical analysis. YZ wrote the first draft of the manuscript. XF and JC wrote sections of the manuscript. All authors contributed to manuscript revision, read and approved the submitted version.

## Conflict of Interest

The authors declare that the research was conducted in the absence of any commercial or financial relationships that could be construed as a potential conflict of interest.
