# Supplementary material for: Validation of 10-Year Stroke Prediction Scores in a Community-Based Cohort of Chinese Older Adults
Source: Front Neurol. 2020 Oct 22;11:986. doi: 10.3389/fneur.2020.00986 (PMC7642878; doi:10.3389/fneur.2020.00986)

Supplementary Material

Supplementary Table 1 | Comparisons of Risk Factors used in the R-FSRS and China-PAR models.

| Revised Framingham Stroke Risk Score | China-PAR stroke risk equations |
| --- | --- |
| Age | Age |
| Systolic blood pressure | Systolic blood pressure |
| Use of antihypertensive medications | Use of antihypertensive medications |
| Diabetes mellitus | Diabetes mellitus |
| Current cigarette smoking | Current cigarette smoking |
| History of CVD | TC |
| Atrial fibrillation | HDL-C |
|  | Waist circumference |
|  | Region |
|  | Urban |
|  | Parental history of stroke |

R-FSRS, revised Framingham Stroke Risk Scores; China-PAR, Prediction for ASCVD Risk in China; CVD, cardiovascular disease; TC, total cholesterol; HDL-C, high-density lipoprotein Cholesterol.

**Supplementary Table 2 |** Comparisons of the regression coefficients for components in the R-FSRS and the BLSA study population.

| **Predictor variables** | **Men** | |  | **Women** | |
| --- | --- | --- | --- | --- | --- |
|  | **Coefficient in R-FSRS** | **Coefficient in BLSA study** |  | **Coefficient in China-PAR** | **Coefficient in BLSA study** |
| **Age per 10 yrs** | 0.49716 | 0.457 |  | 0.087938 | 0.358 |
| **Cerrent smoker (1=yes, 0=No)** | 0.47254 | 0.654 |  | 0.51127 | 0.635 |
| **Prevalent CVD** | 0.45341 | -0.044 |  | -0.03035 | 0.309 |
| **Prevalent AF** | 0.08064 | N/A |  | 1.20720 | N/A |
| **Age 65+** | 0.45426 | -0.751 |  | 0.039796 | -0.154 |
| **DM, if age<65** | 1.35304 | 0.530 |  | 1.07111 | 0.741 |
| **DM, if age 65+** | 0.34385 | 1.064 |  | 0.06565 | -0.538 |
| **HRx** | 0.82598 | -0.157 |  | 0.13085 | 0.365 |
| **SBP per 10, if no HRx** | 0.27323 | 0.121 |  | 0.11303 | 0.111 |
| **SBP per 10, if HRx** | 0.09793 | 0.202 |  | 0.17234 | 0.077 |

R-FSRS, revised Framingham Stroke Risk Scores; China-PAR, Prediction for ASCVD Risk in China; CVD, cardiovascular disease; AF, atrial fibrillation; DM, diabetes mellitus; HRX, hypertension medications; SBP, systolic blood pressure.

**Supplementary Table 3 |** Comparisons of the regression coefficients for components in the China-PAR and the BLSA study population.

| **Predictor variables** | **Men** | |  | **Women** | |
| --- | --- | --- | --- | --- | --- |
|  | **Coefficient in China-PAR** | **Coefficient in BLSA study** |  | **Coefficient in China-PAR** | **Coefficient in BLSA study** |
| **Ln(age), y** | 35.58 | 24.13 |  | 19.97 | -27.52 |
| **Ln(treated SBP), mmHg** | 30.62 | 25.85 |  | 25.87 | -34.74 |
| **Ln(untreated SBP), mmHg** | 29.49 | 23.20 |  | 25.06 | -37.93 |
| **Ln(TC), mg/dL** | 0.29 | -0.77 |  | 0.16 | 1.93 |
| **Ln(HDL-C), mg/dL** | -0.64 | 0.31 |  | -11.35 | 15.92 |
| **Ln(waist circumference), cm** | N/A | N/A |  | 1.60 | -2.07 |
| **Cerrent smoker (1=yes, 0=No)** | 4.72 | -15.44 |  | 0.51 | 0.66 |
| **DM, (1=yes, 0=No)** | 0.30 | 1.11 |  | 0.50 | -0.18 |
| **Geographic region (1=Northern China, 2=Southern China)** | 0.32 | N/A |  | 0.50 | N/A |
| **Urbanization (1=Urban, 0=Rural)** | -0.38 | -1.13 |  | -0.23 | -1.62 |
| **Parent history of stroke (1=Yes, 0=No)** | 7.56 | N/A |  | N/A | N/A |
| **Ln(age)×Current Smoker** | -1.10 | 3.77 |  | N/A | N/A |
| **Ln(age) ×Ln(treated SBP)** | -6.66 | -5.52 |  | -5.77 | 8.55 |
| **Ln(age) ×Ln(untreated SBP)** | -6.40 | -4.9 |  | -5.59 | 9.28 |
| **Ln(age) ×Parental history of stroke** | -1.84 | N/A |  | N/A | N/A |
| **Ln(age) ×Ln(HDL-C)** | N/A | N/A |  | 2.75 | -3.99 |

China-PAR, Prediction for ASCVD Risk in China; BLSA, Beijing Longitudinal Study of Aging; SBP, systolic blood pressure; TC, total cholesterol; HDL-C, high-density lipoprotein Cholesterol; DM, diabetes mellitus.

**Supplementary Table 4 |** Comparisons of baseline characteristics of study

participants included and excluded in final analysis.

| Characteristics | Participants Included  (n=1203) | Participants Excluded*  (n=815) | P value |
| --- | --- | --- | --- |
| Age, mean (SD), year | 68.63 (7.62) | 71.33 (8.12) | <0.001 |
| Male, n (%) | 537 (44.6) | 402(49.3) | 0.038 |
| SBP, mean (SD), mmHg | 138.7 (19.99) | 139.95 (20.26) | 0.187 |
| Waist circumference, mean(SD), cm | 90.8 (10.67) | 90.62 (10.64) | 0.662 |
| Urban, n(%) | 800 (66.5) | 593 (72.8) | 0.003 |
| Antihypertensive treatment, n (%) | 464 (38.6) | 299 (36.7) | 0.392 |
| Diabetes, n (%) | 194 (16.1) | 82 (10.1) | <0.001 |
| Smoking, n (%) | 296 (24.6) | 213 (26.1) | 0.437 |
| History of CVD, n (%) | 187 (15.5) | 133 (16.3) | 0.640 |

SBP, systolic blood pressure; CVD, cardiovascular disease.

**Supplementary Figure 1 |** Flow chart of the BLSA participants included in final analysis.


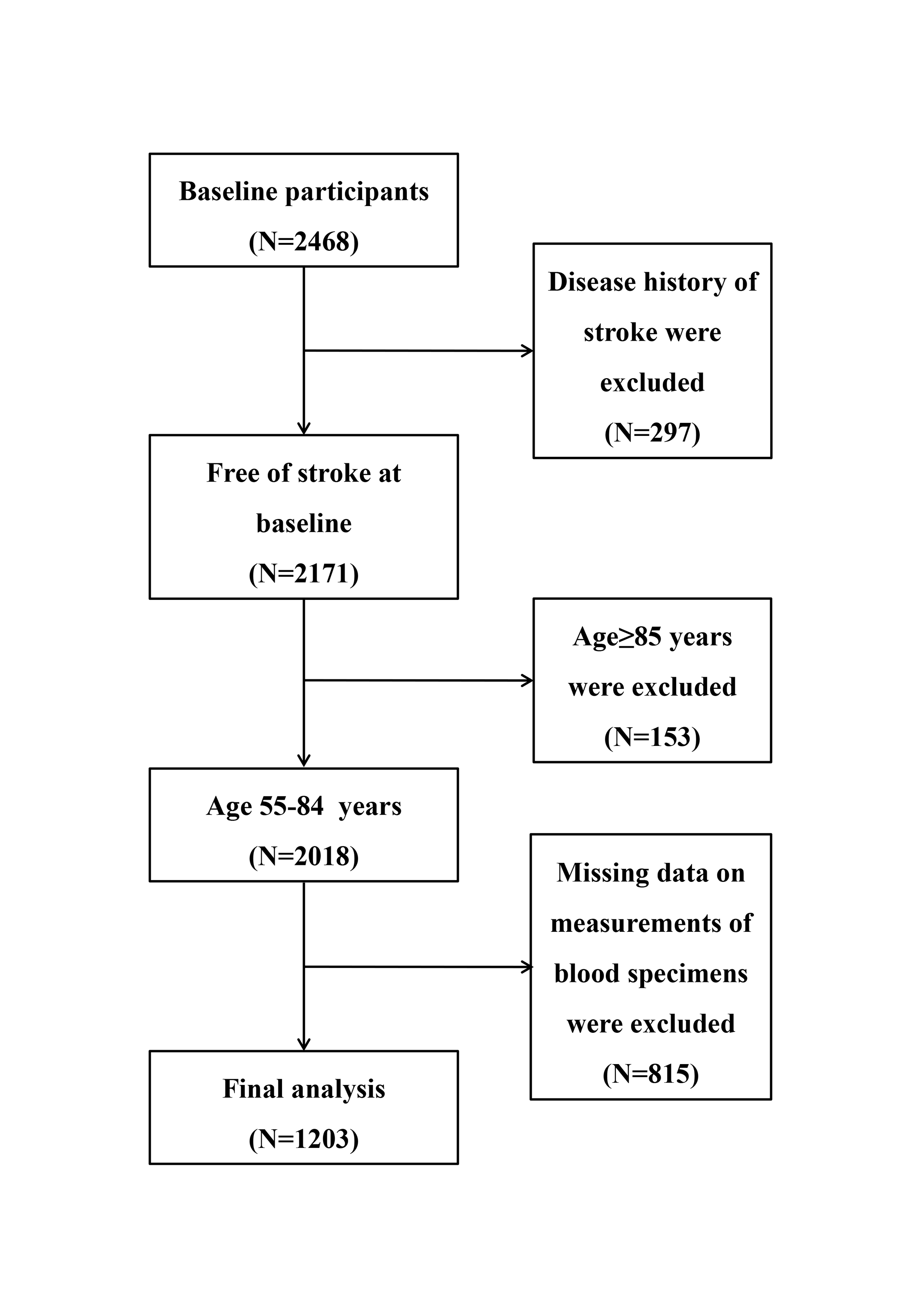

Supplement: Supplementary file 1 [file Data_Sheet_1.doc]
